# Comparison of the Long-term Outcomes of RYGB and OAGB as Conversion Procedures After Failed LSG — a Case–Control Study

**DOI:** 10.1007/s11605-022-05395-w

**Published:** 2022-07-05

**Authors:** Maciej Wilczyński, Piotr Spychalski, Monika Proczko-Stepaniak, Justyna Bigda, Michał Szymański, Małgorzata Dobrzycka, Olga Rostkowska, Łukasz Kaska

**Affiliations:** Department of General, Endocrine and Transplant Surgery, Faculty of Medicine, Medical University of Gdańsk, ul. Smoluchowskiego 17, 80-214 Gdansk, Poland

**Keywords:** Bariatric surgery, Sleeve gastrectomy, Gastric bypass

## Abstract

**Objective:**

To compare the effect of RYGB and OAGB on patients after failed treatment of obesity by laparoscopic sleeve gastrectomy (LSG).

**Study Design:**

A case–control study based on a prospectively maintained database of reoperated patients after failed LSG, which included 33 patients who underwent RYGB conversion and 47 patients who underwent OAGB conversion.

**Result:**

The mean %EBWL after a 5-year follow-up for RYGBc vs OAGBc was 84.04% vs 72.95% (*p* = 0.2176), respectively. Complete long-term diabetes remission was observed significantly more frequently in the OAGBc than in the RYGBc group (97.3% vs 33%; *p* = 0.035). There were no other statistically significant differences in the remission rate of comorbidities between RYGBc and OAGBc: hypertension 30% vs 27.3% (*p* = 0.261), dyslipidemia 83.3% vs 59.1% (*p* = 0.277), OSAS 100% vs 60% (*p* = 0.639), and GERD 40% vs 71.4% (*p* > 0.99), respectively. 7 patients were newly diagnosed with GERD after OAGBc and none after RYGBc. There were no statistically significant differences in the number of complications between the OAGBc and RYGB groups. The Comprehensive Complication Index was 17.85 (± IQR 29.6) in the OAGBc group and 14.92 (± IQR 21.75) in the RYGBc group (*p* = 0.375).

**Conclusion:**

The authors recognized complete long-term type 2 diabetes remission after conversion surgery as the most relevant difference, where the OAGB variety was found superior for its better efficacy. Any other statistically significant differences in the consequences after both conversion procedures used after the failure of LSG have not been stated. Both methods therefore can be considered to complete the initial treatment, considering the preferences and individual burdens of the patients.

## Introduction

The constant demand for bariatric surgery drives its continuous development and has resulted in a wide range of surgical methods available. This variety allows for a personalized, patient-centered approach to the needs of persons with morbid obesity. Still, the most popular procedure is laparoscopic sleeve gastrectomy (LSG), which has had increasing acclaim in recent years.^1^ All bariatric procedures are relatively novel; thus, their complications are still not well known and understood. Moreover, the treatment of such complications is not standardized. Patients after LSG may need secondary surgical treatment due to staple line leaks, unsuccessful weight loss, weight regain, or reflux. In this regard, there is a worldwide tendency to offer this group of patient conversion surgery in the form of a gastric bypass. There are several ways to perform this procedure, but Roux-en-Y gastric bypass (RYGB) and one anastomosis gastric bypass (OAGB) should be distinguished. The former, a widely accepted procedure, has the status of a gold standard of treatment for patients with obesity and concurrent diabetes mellitus. OAGB, on the other hand, as a more recent version of the gastric by-pass, was designed to be simpler and safer, but still is not accepted in all bariatric centers due to possible biliary reflux. Both of these procedures are comparable as a primary procedure.^2–4^ However, there still is a lack of research comparing these two as conversion methods after LSG. Thus, this study aimed to compare the therapeutic effectiveness and safety of RYGB and OAGB performed as conversion surgery after failed LSG.

## Materials and Methods

### Study Subjects

This was a retrospective review of data from a prospectively maintained patient database at the Department of General, Endocrine and Transplant Surgery of the Medical University of Gdansk, Poland, from the years 2009–2020. The following inclusion criteria had to be met: failure or intolerance of LSG treatment for obesity, treated with either RYGB or OAGB, an elective procedure regimen, at least 5 years of regular follow-up at the outpatient clinic of the university obesity center. The qualification, operations, and postoperative observation were conducted by a fixed team of surgeons specializing in bariatric and metabolic surgery. All patients with identified failure of LSG were qualified for a conversion operation. The choice of OAGB versus RYGB procedure as the conversion operation was the joint decision of the patient and the operating surgeon based on the patient’s preference. The primary exclusion criteria from the OAGB group were confirmed Barrett’s esophagus or severe esophagitis (Los Angeles classification C or D).^5^These patients were offered RYGB according to International Federation for the Surgery of Obesity and Metabolic Disorders (IFSO) recommendations^6^ and are included in the RYGB conversion group.

**Fig. 1:**
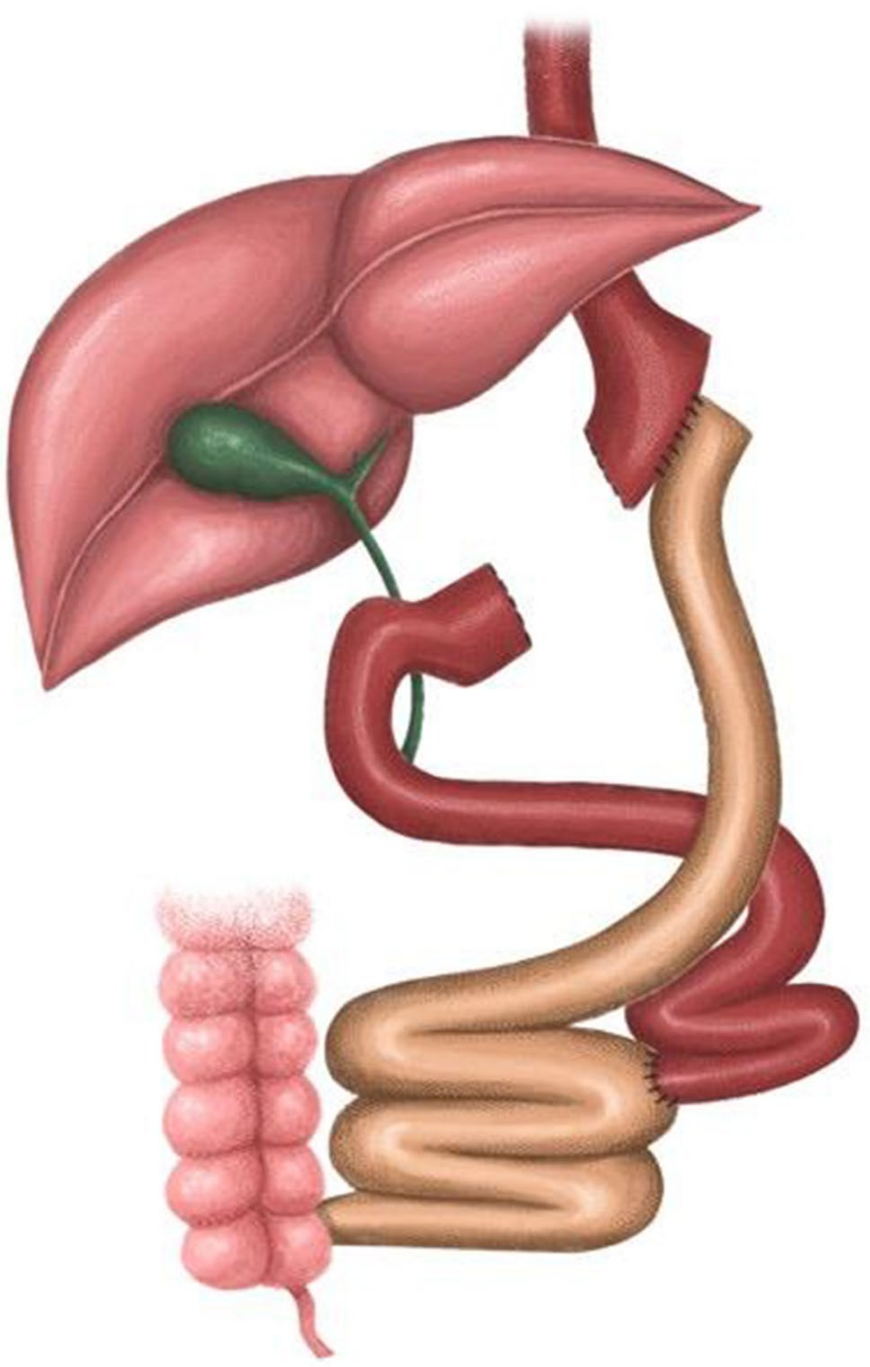
Roux-en-Y gastric bypass conversion

### Medical Procedures

As part of the preoperative preparation, all patients underwent standard blood testing, an upper gastrointestinal endoscopy, an echocardiogram, and an abdominal ultrasound. Two weeks before surgery, a low-calorie and low-carbohydrate diet was recommended.

#### RYGB Technique^7,8^Fig. 1

**Fig. 2:**
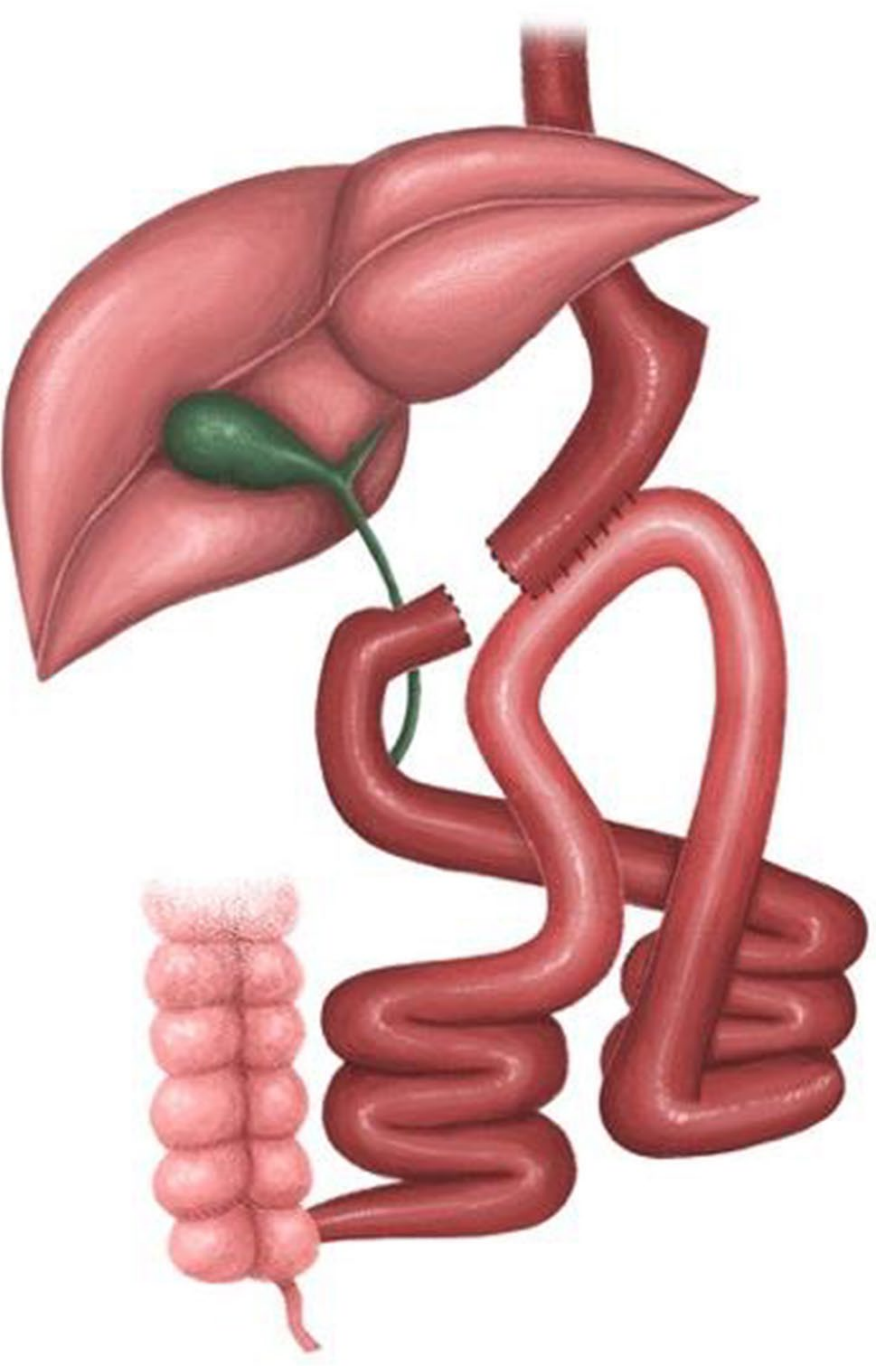
One anastomosis gastric bypass conversion

Our clinical standard includes a transection of the gastric sleeve at the level of the crow’s foot, a biliary-pancreatic limb (BPL) with a length of 100–150 cm, and an alimentary limb (AL) with a length of 100–150 cm. Anastomoses are performed side to side using laparoscopic linear staplers. A 3-cm gastrointestinal anastomosis is located on the anterior wall of the gastric pouch. Gastrointestinal loops are placed anterior to the transverse colon. As a standard, a leak test with methylene blue is per-formed and a closed vacuum drainage is left. The total length of the intestine is not measured, and the mesenteric defects are closed.

#### OAGB Technique^7,8^ Fig. 2

**Fig. 3:**
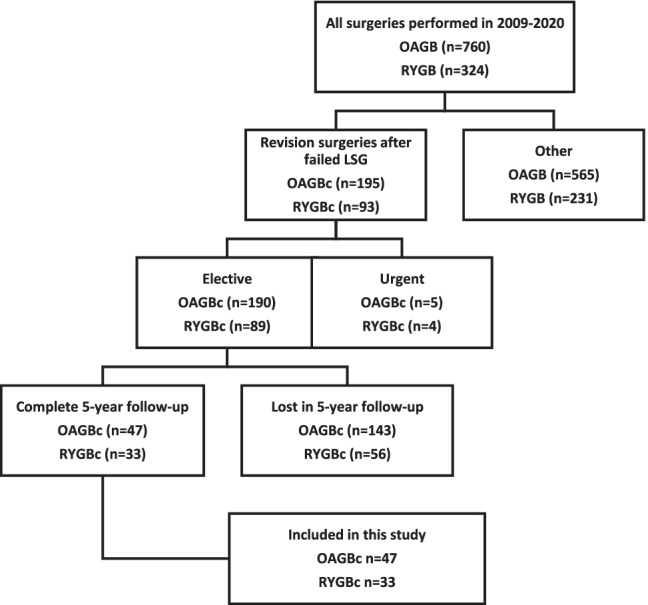
Group enrolment. LSG laparoscopic sleeve gastrectomy, OAGB one anastomosis gastric bypass, RYGB Roux-en-Y gastric bypass, OAGBc OAGB conversion, RYGBc RYGB conversion

OAGB is performed according to our standard by a low transection of the gastric sleeve at the level of the crow’s foot. The length of the BPL is calculated for a length of 180–250 cm. The 3-cm side-to-side anastomosis is performed with a laparoscopic linear stapler. An anti-reflux mechanism is applied in the form of a derotation suture between the distal loop and the stomach. A leak test with methylene blue is performed, and a vacuum drainage is left.

### Follow-Up

Patients were discharged on the first postoperative day with the recommendation to use a liquid diet for 1 week. The diet was extended subsequently to solid foods enriched with protein-rich powder supplements. Thromboprophylaxis in the form of low-molecular-weight heparins was applied for 21 days. Peptic ulcer prophylaxis in the form of 40 mg of pantoprazole per day was recommended for 6 months. Additionally, oral supplementation with a multivitamin tablet and cholecalciferol 4000 IU daily was recommended long-term. The following visits took place in the subsequent postoperative interspace according to the scheme: 1 month, 3 months, 6 months, 1 year, 2 years, 3 years, 4 years, and 5 years. During each visit, a panel of laboratory tests and anthropometric measurements were performed.

### Study Definitions and Outcomes

#### Indications for Conversion — Definition of LSG Failure

The indication for elective surgical conversion to RYGBc or OAGBc was failure of LSG treatment defined as unsatisfactory weight loss (defined when the BMI (body mass index) was over 35 kg/m^2^ and EWL (excessive weight loss) was less than 50%), referring to the Reinhold criteria^9^ or resistant to the medical treatment for symptomatic gastro-esophageal reflux disease after endoscopic evaluation.

### Weight Loss

The assessment was carried out regarding the change of body weight and BMI on subsequent visits. To objectively assess the effects of the obesity treatment, the percentage of excess weight loss (%EWL) calculated from the weight before the first bariatric operation and the percentage of total body weight loss (TWL) calculated from the date of the conversion operation were also evaluated.

#### Type 2 Diabetes Mellitus

Type 2 diabetes mellitus (DM2) was defined according to the Polish guidelines on the management of patients with diabetes.^10^ two independent results of fasting glycemia > 126 mg/dl or glycemia at 120 min OGTT (Oral Glucose Tolerance Test) > 200 mg/dl or HbA1c (Glycated hemoglobin) > 6.5%. Complete DM2 remission was defined as the euglycemic results of laboratory tests (fasting glucose level < 100 mg/dL with HbA1c level < 6.0%) at the discontinuation of insulin therapy and oral antidiabetic drugs. Patients who achieved only partial remission (reached only partial improvement on the fasting glucose level, HbA1c level, reduced doses of insulin and/or oral antidiabetic drugs) were not considered as treatment success in the evaluation of the outcome.

#### Hypertension

Hypertension (HT) was defined as an arterial pressure higher than 140/90 mmHg. The remission of HT was considered to achieve an arterial pressure of < 120/80 mmHg with the simultaneous withdrawal of antihypertensive medicaments.

#### Obstructive Sleep Apnea Syndrome

OSAS was recognized when the AHI (Apnea–Hypopnea Index) > 14 or AHI > 4 with typical symptoms. The follow-up was not controlled by a polysomnographic examination. Remission was considered to be the resolution of the clinical symptoms typical for OSAS.

#### Dyslipidemia

Dyslipidemia (DL) remission was diagnosed by levels of total cholesterol < 200 mg/dl and HDL > 40 mg/dl in men and > 50 mg/dl in women.

#### Nutritional Parameters and Anemia

Nutritional parameters were estimated at each visit to the surgical outpatient clinic. Anemia was diagnosed according to the WHO (World Health Organization) definition, with hemoglobin < 12 g/dl in women and < 13 g/dl in men. Moderate anemia was defined as hemoglobin values of 8–11 g/dl. Severe anemia was defined as hemoglobin at < 8 g/dl. Nutritional status was assessed by determining the levels of albumin (norm 30–50 g/l), iron (norm 50–170 µg/dl), vitamin B12 (norm 180–914 ng/l), and 25-hydroxy vitamin D (norm 10–50 ng/ml).

### Gastro-esophageal Reflux Disease

Gastro-esophageal reflux was diagnosed based on symptoms in the form of regurgitation and a feeling of heartburn despite adequate medical treatment. All patients were evaluated preoperatively with esophagogastroduodenoscopy to confirm endoscopic features of reflux such as esophagitis assessed in Los Angeles classification.^5^ Thereafter, during the postoperative follow-up period, patients were required to undergo follow-up endoscopy after 2 years or sooner in case of symptoms of gastroesophageal reflux. As a standard, neither multichannel intraluminal impedance nor pH-metry were not performed. Remission was defined as the complete resolution of clinical symptoms and independence from oral anti-reflux medications.

### Outcomes and Outcome Measures

The primary outcome was the results of weight loss over a 5-year period after the conversion surgery, measured as a change of %EWL and %TWL. The secondary outcome was the effectiveness of methods in the treatment of comorbidities, measured as the ratio of comorbidities after 5 years. The tertiary outcome was the safety of both methods, measured as the rate of complications. The goal was to indicate a method that would give better weight loss results 5 years after the conversion operation. The secondary goal was to indicate the method characterized by a more effective remission of comorbidities. The tertiary goal was to indicate the method characterized by a safer profile of complications.

### Statistical Methods

Descriptive statistics were performed using means and standard deviations (SDs) for normally distributed data, and medians and interquartile ranges (IQRs) for the remaining data. Normality was tested with the Shapiro–Wilk test. Categorical data were compared with the use of contingency tables and chi-squared tests, and if there were less than 5 observations, Fisher’s exact test was used. Continuous data with normal distributions were compared with *t*-tests, and for samples with unequal variance, Welch’s correction was used. Continuous data with non-normal distribution were compared with Mann–Whitney *U* tests. The *P*-value was considered significant at < 0.05. All tests were two-sided. All analyses were performed, and graphs were plotted with the use of GraphPad Prism 8.4.3 (GraphPad Software, LLC., CA, USA) statistical software.

## Results

### Study Sample

Out of 3140 patients treated surgically in the Department of General, Transplant and Endocrine Surgery between 2009 and 2020, two groups of patients were initially distinguished who underwent RYGB (*n* = 93) or OAGB (*n* = 760) surgery. Out of these, 33 patients after RYGB and 47 after the OAGB procedure met the inclusion criteria. The enrolment process is shown in Fig. 3. Comparative demographic data are presented in Table 1. In the study group, the reasons for conversion were unsatisfactory weight loss in 44 (93.6%) OAGBc and 20 (60.6%) RYGBc patients, weight regain in 2 (4.3%) OAGBc and 5 (15.2%) RYGBc patients, and symptomatic reflux in 1 (2.1%) OAGBc and 8 (24.2%) RYGBc patients. In the preoperative endoscopic evaluation of patients qualified for conversion due to reflux, a patient in the OAGBc group presented with heartburn and regurgitations without features of esophagitis. Otherwise, in the RYGBc group, there were six cases of grade C esophagitis and one grade D esophagitis. No patient in the study group had Barrett’s esophagus. Patients with reflux burden but qualified for conversion for other reasons had no evidence of esophagitis on preoperative gastroscopy.

**Table 1:** Demographic characteristics before conversion. OAGBc – one anastomosis gastric bypass conversion. RYGBc – Roux-en-Y gastric bypass conversion. BMI – body mass index. WBC – white blood count. DM2 – diabetes mellitus type 2. HT – hypertension. OSAS – obstructive sleep apnea syndrome. DL – dyslipidemia. GERD – gastroesophageal reflux disease

	RYGBc (*n* = 33)	OAGBc (*n* = 47)	*p* value
Age (mean ± SD)	41.24 ± 8.906	45.02 ± 10.71	0.100
Sex (F/M)	27 / 6	34 / 13	
Weight ± SD (kg)	105.52 ± 18.1	115.17 ± 20.81	0.036
BMI ± SD (kg/m^2^)	38.70 ± 6.84	40.44 ± 5.8	0.228
Albumin ± IQR (mg/dl)	38 ± 3	39.79 ± 3	0.146
Creatinine ± IQR (mg/dl)	0.75 ± 0.1	0.8 ± 0.15	0.033
Hemoglobin ± SD (g/dl)	13.5 ± 1.22	14.37 ± 1.3	0.012
WBC ± SD (10′3/ul)	7.331 ± 1.77	7.52 ± 1.53	0.658
DM2 *n* (%)	6 (18%)	12 (25.5%)	0.438
HT *n* (%)	10 (30%)	22 (47%)	0.138
OSAS *n* (%)	1 (3%)	5 (11%)	0.203
DL *n* (%)	6 (18%)	22 (47%)	0.008
GERD n (%)	10 (30%)	7 (15%)	0.097
Conversion reason			
Unsatisfactory weight loss	20 (60.6%)	44 (93.6%)	
Weight regain	5 (15.2%)	2 (4.3%)	
Symptomatic reflux	8 (24.2%)	1 (2.1%)	

### Weight Loss

**Fig. 4:**
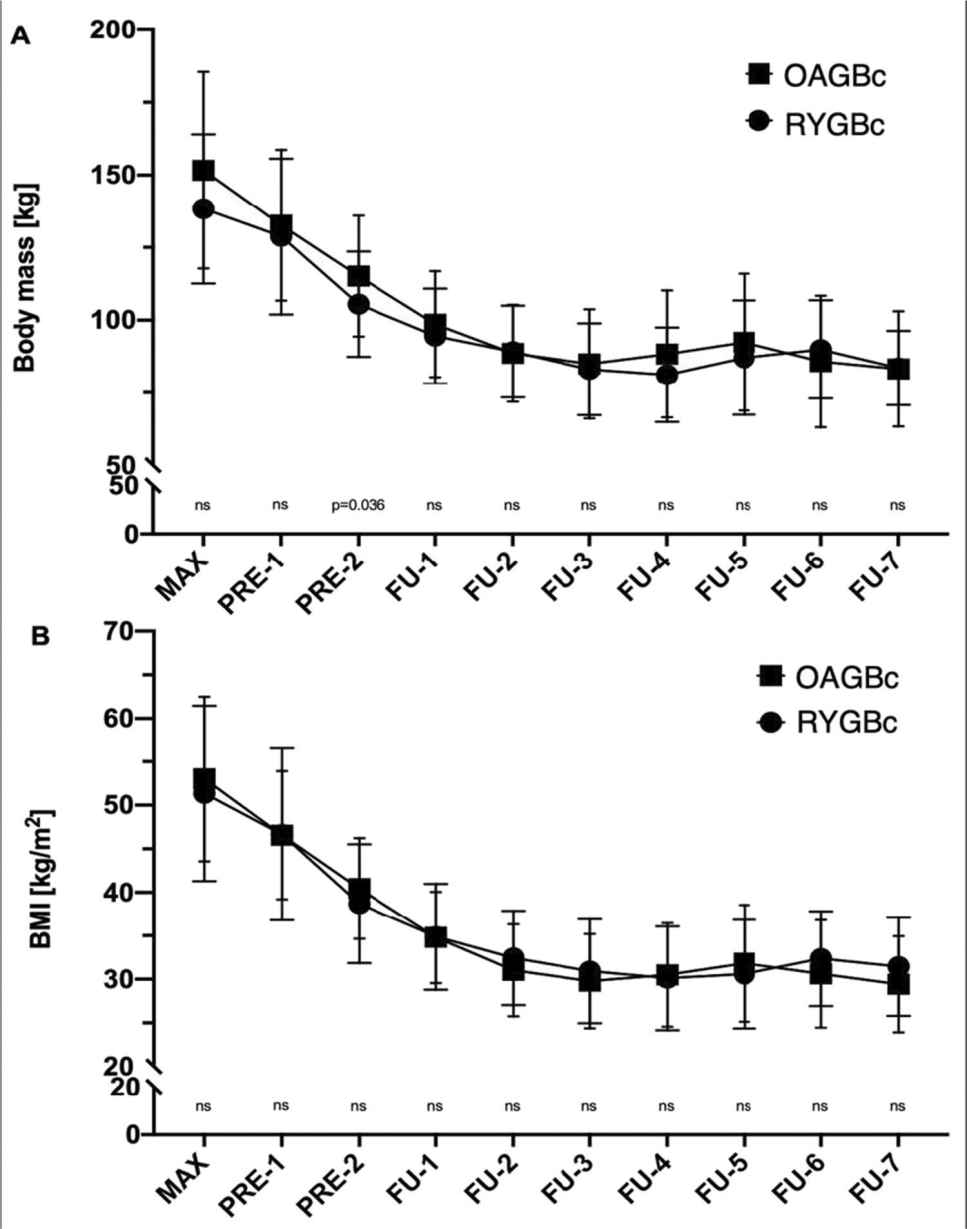
Weight (panel **A**) and BMI (panel **B**) changes: means with SDs. Significant *p*-values are shown. Non-significant *p*-values are shown as “ns.” SDs for RYGBc have long dashes. SDs for OAGBc have short dashes. Note that the *Y*-axis has been divided to better visualize details. OAGBc one anastomosis gastric bypass conversion, RYGBc Roux-en-Y gastric bypass conversion, MAX maximum weight/BMI, PRE-1,2 weight/BMI before conversion. Consecutive follow-up: FU1 — 3 months, FU-2 — 6 months, FU-3 — 1 year, FU-4 — 2 years, FU-5 — 3 years, FU-6 — 4 years, FU-7 — 5 years

After conversion surgery, no statistically significant differences in weight loss and BMI were found between the RYGBc (RYGB conversion) and OAGBc (OAGB conversion) groups except for a higher %TWL after 4 years of follow-up in the OAGBc group than in the RYGBc group (25.17 ± 11.87% vs 13.4 ± 9.38% *p* = 0.018, respectively). Full results of %EWL and %TWL are shown in Table 2. Changes in weight loss and BMI are shown in Fig. 4.

**Table 2:** Weight loss. OAGBc – one anastomosis gastric bypass conversion. RYGBc – Roux-en-Y gastric bypass conversion. EWL% – percentage of excess weight loss. TWL% – percentage of total body weight loss

	OAGBc	RYGBc	*p *value
EWL% after 1 y ± SD (%)	85.1 ± 18.68	79.47 ± 21.15	0.302
EWL% after 2 y ± SD (%)	82.57 ± 21.48	79.6 ± 22.99	0.666
EWL% after 3 y ± SD (%)	78.64 ± 24.85	73.51 ± 23.33	0.578
EWL% after 4 y ± SD (%)	82.86 ± 19.32	70.43 ± 17.19	0.121
EWL% after 5 y ± SD (%)	84.04 ± 18.81	72.95 ± 20.3	0.218
TWL% after 1 y ± SD (%)	26.78 ± 10.09	21.48 ± 11.27	0.073
TWL% after 2 y ± SD (%)	25.66 ± 11.2	19.76 ± 14.34	0.128
TWL% after 3 y ± SD (%)	24.46 ± 13.83	13.85 ± 13.85	0.050
TWL% after 4 y ± SD (%)	25.17 ± 11.87	13.4 ± 9.38	0.018
TWL% after 5 y ± SD (%)	21.81 ± 12.48	18.39 ± 11.85	0.546

### Impact on Comorbidities

After a 5-year follow-up period, DM2 remission was reached significantly more often in OAGBc (11/12; 91.7%) than in RYGBc (2/6; 33%) patients (*p* = 0.035). GERD remission was reached in 5 (71.4%) OAGBc and 4 (40%) RYGBc patients (*p* > 0.99) with complete resolution of reflux symptoms and features of esophagitis in endoscopy. In the RYGBc group, of the 10 patients with GERD before conversion, 4 patients achieved complete remission — these were patients qualified for conversion due to GERD. The remaining 6 patients achieved a reduction in their symptoms and a reduction in esophagitis to LA grade A. There were no new cases of GERD after conversion in this group. In the OAGBc group of 7 patients with GERD before conversion, 5 achieved complete remission — including the one patient who was eligible for conversion because of GERD. In the other two patients, symptoms of GERD persisted with no endoscopic features of esophagitis before or after conversion. However, there were 7 new cases of symptomatic GERD without features of esophagitis in endoscopy in this group after conversion. Changes in the prevalence of the remaining comorbidities did not differ significantly after the conversion operation and are presented in detail in Table 3.

**Table 3:** Comorbidities. LSG – laparoscopic sleeve gastrectomy. OAGB – one anastomosis gastric bypass. RYGB – Roux-en-Y gastric bypass. BMI – body mass index. WBC – white blood count. DM2 – diabetes mellitus type 2. HT – hyper-tension. OSAS – obstructive sleep apnea syndrome. DL – dyslipidemia. GERD – gastroesophageal reflux disease

Comorbidities	RYGB (*n* = 33)	OAGB (*n* = 47)	*p* value
DM2 before LSG *n* (%)	10 (30%)	16 (34%)	0.811
DM2 before conversion	6 (18%)	12 (25.5%)	0.588
DM2 after conversion	4 (12%)	1 (2%)	0.026
DM2 remissions *n* (%)	2 (33%)	11 (91.7%)	0.035
HT before LSG	13 (39%)	25 (53%)	0.261
HT before conversion	10 (10%)	22 (47%)	0.168
HT after conversion	7 (21%)	16 (34%)	0.3157
HT remissions *n* (%)	3 (30%)	6 (27.3%)	0.729
OSAS before LSG	3 (9%)	5 (11%)	> 0.999
OSAS before conversion	1 (3%)	5 (11%)	0.392
OSAS after conversion	0	2 (4%)	0.509
OSAS remissions *n* (%)	1 (100%)	3 (60%)	0.639
DL before LSG	6 (18%)	23 (49%)	0.005
DL before conversion	6 (18%)	22 (47%)	0.010
DL after conversion	1 (3%)	9 (19%)	0.041
DL remissions *n* (%)	5 (83.3%)	13 (59.1%)	0.277
GERD before LSG	5 (15%)	8 (17%)	> 0.999
GERD before conversion	10 (30%)	7 (15%)	0.16
GERD after conversion	6 (18%)	9 (19%)*	> 0.999
GERD remissions *n* (%)	4 (40%)	5 (71.4%)	> 0.999

^*^7 newly diagnosed cases of reflux not seen before conversion

### Impact on Nutritional Status

During the 5-year follow-up period, 22 (46.8%) OAGBc patients and 14 (42.4%) RYGBc patients (*p* = 0.698) developed anemia. In all cases, it was microcytic anemia treated with additional oral iron supplementation, except for two patients requiring intravenous supplementation (one from each group). The details are presented in Table 4. No statistically significant differences in hemoglobin concentrations between patients after OAGBc and RYGBc were found. No statistically significant differences between the groups were detected in the concentrations of iron, vitamin B12, vitamin D, and serum albumin in subsequent years. The results are presented in Table 5.

**Table 4:** Anemia and hemoglobin levels. OAGBc – one anastomosis gastric bypass conversion. RYGBc – Roux-en-Y gastric bypass conversion. Hb – hemoglobin serum level

Anemia	OAGBc *n* = 47	RYGBc *n* = 33	*p* value
Mild anemia	9	8	0.592
Moderate anemia	12	6	0.588
Severe anemia	1	0	> 0.999
Anemia general	22 (46.8%)	14 (42.4%)	0.352
Hb level before LSG ± SD (g/dl)	14.54 ± 1.31	14.19 ± 0.68	0.471
Hb level before conversion ± SD (g/dl)	14.37 ± 1.3	13.5 ± 1.22	0.012
Hb level after 1 y ± IQR (g/dl)	13.04 ± 1.55	12.69 ± 2.27	0.079
Hb level after 2 y ± SD (g/dl)	12.61 ± 2	12.79 ± 1.82	0.744
Hb level after 3 y ± IQR (g/dl)	12.78 ± 1.68	11.95 ± 1.95	0.116
Hb level after 4 y ± SD (g/dl)	12.73 ± 1.79	11.98 ± 1.88	0.251
Hb level after 5 y ± SD (g/dl)	12.36 ± 1.84	11.82 ± 2.1	0.548

**Table 5:** Nutritional status parameters. OAGBc – one anastomosis gastric bypass conversion. RYGBc – Roux-en-Y gastric bypass conversion

	OAGBc	RYGBc	*p* value
Iron before conversion ± SD (µg/dl)	100.9 ± 34.45	130.2 ± 87.61	0.204
Iron after 1 y ± IQR (µg/dl)	96.14 ± 51.05	106.8 ± 38	0.341
Iron after 2 y ± SD (µg/dl)	75.85 ± 34.29	61.58 ± 32.12	0.215
Iron after 3 y ± IQR (µg/dl)	78.68 ± 57.3	59.3 ± 33.75	0.140
Iron after 4 y ± SD (µg/dl)	81.16 ± 40.6	91.27 ± 54.35	0.539
Iron after 5 y ± SD (µg/dl)	67.05 ± 41.23	67.83 ± 40.43	0.968
Vit. B12 before conversion ± IQR (ng/ml)	490.9 ± 286.2	324.5 ± 132.5	0.109
Vit. B12 1 y ± IQR (ng/ml)	444 ± 238	448.4 ± 430.5	0.953
Vit. B12 2 y ± IQR (ng/ml)	653 ± 307.5	568.3 ± 719.2	0.662
Vit. B12 3 y ± IQR (ng/ml)	2809 ± 274.2	561.3 ± 376	0.614
Vit. B12 4 y ± IQR (ng/ml)	454 ± 180.5	398.2 ± 332.2	0.809
Vit. B12 5 y ± SD (ng/ml)	379.1 ± 155.9	370.5 ± 130	0.905
Vit. D before conversion ± SD (ng/ml)	20.23 ± 9.58	24.68 ± 8.80	0.389
Vit. D 1 y ± IQR (ng/ml)	22.01 ± 14.8	20.44 ± 21.98	0.344
Vit. D 2 y ± SD (ng/ml)	19.84 ± 10.29	27.91 ± 20.32	0.088
Vit. D 3 y ± IQR (ng/ml)	23.49 ± 15	25.16 ± 31	0.964
Vit. D 4 y ± IQR (ng/ml)	22.82 ± 16.7	21.25 ± 15.9	0.525
Vit. D 5 y ± IQR (ng/ml)	39.11 ± 49.95	28.4 ± 22.55	0.545
Albumin before conversion ± IQR (g/l)	39.79 ± 3	38 ± 3	0.146
Albumin 1 y ± IQR (g/l)	37.65 ± 4	37.25 ± 5.5	0.522
Albumin 2 y ± IQR (g/l)	37.88 ± 4	38.15 ± 6	0.496
Albumin 3 y ± IQR (g/l)	37.15 ± 3.38	36.33 ± 6.8	0.702
Albumin 4 y ± IQR (g/l)	36 ± 3.75	36.44 ± 7	0.668
Albumin 5 y ± SD (g/l)	37.21 ± 3.58	40.33 ± 1.97	0.055

### Complications

There were no statistically significant differences in the number of complications between the OAGBc and RYGBc groups. The Comprehensive Complication Index (CCI®)^11^ was 17.85 (± IQR 29.6) on average in the OAGBc group and 14.92 (± IQR 21.75) in the RYGBc group at *p* = 0.375. Symptomatic GERD occurred in 6 patients after OAGBc and 3 patients after RYGBc. Upper gastrointestinal bleeding occurred in two patients of each group. Marginal ulceration of the gastrointestinal anastomosis was diagnosed in 3 OAGBc and 4 RYGBc patients. Perforation of the anastomotic site occurred in 3 (6.3%) patients of the OAGBc but none from the RYGBc group (respectively after 3, 3, and 5 years post OAGBc). The first case was a patient with a history of gastritis in whom the obstetrician discontinued proton pump inhibitors during pregnancy, the perforation occurred a few months after delivery, and the patient did not undergo the recommended endoscopic follow-up after delivery. The second case was a patient who developed anastomotic perforation during antiviral treatment for hepatitis C. The third case involved a perforation of the anastomotic site on the first day after cholecystectomy of unclear iatrogenic or ulcerative etiology. No early anastomotic leaks (< 30 day post OAGBc/RYGBc) were identified. Stenosis of the gastrointestinal anastomosis was found in two patients from each group. One patient from the OAGBc and none from the RYGBc group experienced a perforation of the small intestine due to iatrogenic injury during abdominoplasty. Hemoperitoneum was found in one patient after RYGBc and in none of the OAGBc patients. An infection of the surgical wound was found in two patients of each group. Rehospitalization due to abdominal pain was necessary for two OAGBc patients and one RYGBc patient. Symptomatic malnutrition syndrome was found in 4 OAGBc patients and 3 RYGBc patients. Only the patient with a perforation of the small intestine required reoperation. Perforations of the gastrointestinal anastomosis site were effectively treated endoscopically with a gastric stent. An analysis of the frequency and severity of complications is presented in Tables 6 and 7.

**Table 6:** Complications. OAGBc – one anastomosis gastric bypass conversion. RYGBc –Roux-en-Y gastric bypass conversion. GERD – gastroesophageal reflux disease. GI – gastro-intestinal

	OAGBc	RYGBc	*p*	Clavien-Dindo
GERD	6	3	0.729	2
Anemia	22	14	0.352	2
GI bleeding	1	1	> 0999	3a
Hematoperitoneum	0	1	0.413	2
Malnutrition	4	3	> 0.999	2
Anastomosis ulceration	3	4	0.439	2
Anastomosis stenosis	1	1	> 0.999	3a
Abdominal pain	2	1	> 0.999	1
Anastomosis perforation	3	0	0.264	3b
Bowel perforation	1	0	> 0.999	4b
Wound infection	1	1	> 0.999	3b

**Table 7:** Clavien-Dindo scoring

Clavien-Dindo	OAGBc	RYGBc	*p*
I	2	1	0.043
II	35	25	0.13
IIIa	2	1	0.043
IIIb	6	1	0.128
IVa	0	0	0.000
IVb	1	0	0.21
V	0	0	0.000

## Discussion

Our study did not reveal statistically significant differences in weight loss between the RYGBc and OAGBc operations. Both evaluated procedures are also characterized by a similar course of postoperative BMI change, in which a rapid decline in the first year after surgery and a slight increase and stabilization in the third year of observation has been noticed. These results show that both bypass methods of conversion allow the criteria of the effectiveness of bariatric treatment to be achieved with a similar stability profile; however, it was not possible to indicate the more effective one. This result may correspond to the results of the primary operations obtained in the YOMEGA study^2^ in which %EWL of 87.9% in RYGB patients and 85.8% in OAGB patients after a 2-year follow-up period and found no significant statistical difference between these procedures. A similar lack of differences was published by Lee et al. in 2005^12^ after a 2-year follow-up period. Regarding the remission of diseases accompanying obesity, the potential advantage of OAGBc over RYGBc was demonstrated in the treatment of diabetes. A potential explanation could be that the high concentration of bile in a distinctly long BPL promotes the enhancement of the portal recirculation of bile acids, which eventually influences metabolism improvement.^13^ Similar results were obtained in a weighty YOMEGA^2^ study and a meta-analysis published by Magouliotis et al.^14^ suggesting that patients with DM2 may benefit more from OAGB review surgery.

GERD is often the reason for qualification for conversion after LSG. It has been evidenced to increase the incidence of esophagitis and Barrett’s esophagus.^15^ The beneficial effect found in our study on the resolution of esophagitis in patients after RYGBc is confirmed by similar Felsenreich et al. findings.^16^ The limitation of our study is the lack of analysis of the effect of OAGBc on esophagitis. It resulted from following the IFSO recommendations^6^ from the period in which the patients were qualified for conversion. RYGBc was recommended method in patients with esophagitis following LSG. However, the analysis of the effect of conversion to gastric bypass on the treatment of reflux is very difficult. This is mainly because both acid and biliary reflux have similar symptoms, and tests that distinguish between them, e.g., multichannel intraluminal impedance (MII), have only recently started to appear in the standards of care for bariatric patients. First study analyzing this indicates an increased incidence of biliary reflux after OAGB assessed in MII.^17^ Thus, it may be the cause of newly diagnosed GERD in patients in the OAGBc group. However, its role in the persistence of reflux in RYGBc patients should also be considered. A more detailed analysis of the prevalence of biliary reflux and its impact on patients after gastric bypass surgery needs to be further investigated in future studies. The concern regarding the oncological consequences of biliary reflux^18,19^ , still is widely debated on international forums.^20^

With regard to the risk of developing anemia and malnutrition complications following our proposed conversion options, no statistically significant differences have been found. Studies suggesting more frequent deficiency complications after OAGB surgery, such as by Genser et al.^21^ , are based on the analysis of patients with an average BPL length of 320 cm. On the basis of these results, a simple correlation between the length of the BPL and the risk of deficiency complications can be suspected. How-ever, in the YOMEGA^2^ study, when comparing 200 cm BPL–OAGB to RYGB with 150 cm AL and 50 cm BPL, a statistically significant difference in the incidence of malnutrition complications was reported, exposing OAGB as the more hazardous procedure. This may indicate that the mechanism of occurrence of such distant complications is still insufficiently studied and requires a deeper analysis; however, the standardization of the procedures should first be established. The guidelines for reporting the technical details described in publications on bariatric operations also should be unified. The education and compliance of patients in the postoperative long-term period are commonly approved as being the fundamental independent factor influencing the effect of primary and conversion surgeries.^22, 23, 24^ Although the authors’ institution provides postoperative psychological and dietetic care, the remote measurement of compliance with diet and supplementation seems to be extremely difficult. However, continuous monitoring of the nutritional status of post-bariatric patients is the obvious duty of the multidisciplinary team.

The percentage of complications found in the study group appears to be high. They are coherent with the fact stated in a large multicenter analysis that conversion surgery is burdened with a higher rate of complications and perioperative mortality than primary surgery.^25^ However, as in our material, most of them can be successfully treated with endoscopy. It is noteworthy that most late surgical complications arise from ulceration of the anastomotic region. The risk of ulcer formation seems to accompany patients after gastric bypass surgery for the rest of their lives. Therefore, it is important to pay attention to endoscopic follow-up and prompt treatment of diagnosed peptic ulcer disease.

The limitations of the present study include the retrospective and observational character of the collected data. The database, however, was constructed and has been maintained prospectively and contains the parameters needed for the study. A relatively small study sample is the result of a combination of the long follow-up and unsatisfactory compliance to the follow-up scheme. The latter may be a trait typical for this group of patients.^26^ The observed loss to follow-up after 5 years was 74.5% for OAGBc and 60.7% for RYGBc. In accordance, Higa et al. reported a follow-up rate after RYGB of 33% at 2 years and 7% at 10 years.^27^ During the procedures, the total intestinal length was not measured, which is the standard of care at our Institution and is confirmed in the IFSO recommendations.^7^ Bias may also have occurred at the time of qualification. The data in Table 1 shows that the group of patients eligible for conversion due to the GERD was qualified for RYGBc. It was probably due to the willingness to follow the IFSO recommendations of the time and concern about overlapping consequences of bile reflux with acid reflux. That caused GERD with severe esophagitis to be an exclusion criterium from the OAGBc group. Nevertheless, OAGBc also presents an anti-reflux effect against acid reflux, specifically when improved with one of the anti-reflux mechanisms.^28^ An independent comparison of the treatment of this complication of LSG would require a randomized controlled, blinded trial that could reliably answer the question regarding the superiority of either method. Furthermore, the low number of diabetes cases may bias the achieved outcome. The remission of diabetes depends on multiple factors that are not assessed in this study, such as age, sex, duration of the disease, drug treatment with oral hypoglycemic agents or insulin, and pancreatic reserve. Similarly, due to the lack of initial data regarding nonalcoholic fatty liver disease and liver function parameters, we were not able to evaluate these outcomes. Furthermore, the standard of care in the years of the observation period did not include an assessment of compliance with following the recommended vitamin supplementation regimen, and indicators of the change in the quality of life after surgery. The lack of significant differences in weight loss and BMI change between analyzed groups may also be due to the aforementioned retrospective nature and limited study sample, and therefore, these results should be interpreted with care. Some of the presented analyses may have been underpowered to detect significant changes. However, due to the specific clinical scenario that was analyzed and a relatively long follow-up, we believe that this study adds to the state of knowledge despite the limitations listed above.

## Conclusion

Based on the results obtained in our study, OAGBc is an equally effective procedure as RYGBc for patients after the failure of LSG treatment. Both procedures demonstrate a similar safety profile and weight loss effect. OAGBc can also bring potential additional benefits to diabetic patients. Unfortunately, the consequences of reflux complications, which are more likely to affect patients during the follow-up period after OAGB, require further study.
